# Changes in Photosynthetic Pigments, Total Phenolic Content, and Antioxidant Activity of *Salvia coccinea* Buc’hoz Ex Etl. Induced by Exogenous Salicylic Acid and Soil Salinity

**DOI:** 10.3390/molecules23061296

**Published:** 2018-05-29

**Authors:** Monika Grzeszczuk, Piotr Salachna, Edward Meller

**Affiliations:** 1 Department of Horticulture, West Pomeranian University of Technology, 3 Papieża Pawła VI Str., Szczecin 71-459, Poland; monika.grzeszczuk@zut.edu.pl; 2 Department of Soil Science, Grassland Management and Environmental Chemistry, West Pomeranian University of Technology, Słowackiego 17 Str., Szczecin 71-434, Poland; edward.meller@zut.edu.pl

**Keywords:** scarlet sage, salicylic acid, NaCl, polyphenols, FRAP, ABTS

## Abstract

*Salvia coccinea* (Lamiaceae) is a promising source of potential antioxidants, and its extracts can be used in pharmaceutical industry, as well as in food products and cosmetics. Salicylic acid (SA) affects many physiological and metabolic processes in vascular plants under salinity stress. The aim of this study was to investigate the response of *S. coccinea* to either SA, or sodium chloride (NaCl), or a combination of both. The plants were sprayed with a solution of 0.5 or 1.0 mM SA and watered with 0, 100, 200, or 300 mM NaCl. Exogenous application of SA increased the number of branches, fresh herbal weight, and total chlorophyll content vs control plants. Salinity-exposed plants showed reduced growth, content of photosynthetic pigments total polyphenols, and antioxidant activity. However, foliar application of SA relieved the adverse effects of 100 mM NaCl, as demonstrated by increased number of branches, greater fresh herbal weight, higher content of total chlorophyll, total carotenoids, and total polyphenols, as well as antioxidant potential, detected using ferric-reducing ability of plasma (FRAP) and 2.2′-azino-bis(3-ethylbenzothiazoline-6-sulfonic acid) diammonium salt (ABTS), compared with untreated plants.

## 1. Introduction

*Salvia* genus belongs to Lamiaceae family and includes approximately 900 species widely distributed throughout the world [1,2]. Many of them have been used for centuries in traditional medicine, as antihydrotics, tonics, antirheumatoids, antimicrobials, antispasmodics, antiseptics, astringents, digestives, anti-inflammatories, carminatives, and chronic painkillers, and also as a spice for flavoring and preserving foods, and in cosmetics and perfumes [3,4,5]. Several studies identified *Salvia* genus as one of the most valuable sources of natural antioxidants [6,7]. The main chemical components identified in sage are essential oils, hydroxycinnamic acid derivatives, phenolic diterpenes, triterpenes, flavonoids, phenolic glycosides, and polysaccharides [3,8]. Carnosol and carnosic acid (phenolic diterpenes), rosmarinic acid, caffeic acid, ferulic acid and chlorogenic acid (hydroxycinnamic acid derivatives), salvianolic acid (dimer of rosmarinic acid), and also some essential oil compounds (camphor, 1, 8-cineole, α-thujone, β-thujone) and flavonoids (flavones of apigenin and luteolin, and their hydroxylated derivates) were identified as the major antioxidants present in sage [4,7,9]. These antioxidants help in maintaining health, and protect against atherosclerosis, stroke, diabetes, neurodegenerative diseases, and cancer. Their properties made them highly interesting to scientists, food manufacturers, and consumers [10,11,12].

The high food demand of growing human population requires a reduction of the harmful effects of salinity that is as one of the major factors affecting plant crop productivity worldwide [13,14,15]. Using exogenous salicylic acid (SA) for this purpose has been proposed by many scientists [16,17]. SA is a phytohormone (phenolic-type regulator, a non-enzymatic antioxidant) and the most biologically active mediator of plant biotic and abiotic stress responses [18,19,20]. It affects plant growth and development, seed germination, thermogenesis, stomatal regulation and photosynthesis, ion uptake, as well as antioxidant activity [21,22,23]. Exogenous application of SA enhances tolerance to salt stress in several medicinal plant species [24,25]. However, plant response to SA may differ even within the same species, and depends on its dose and application method, plant age, its nutritional status, and environmental conditions [26]. Sahar et al. [27] reported that salicylic acid treatment increased proline accumulation in the leaves of *Salvia officinalis* grown under salinity stress, and increasing levels of proline are known to boost resistance to salt stress. The mechanisms of the beneficial effects of SA application in sage remain largely unknown. 

One of the more interesting sages is *Salvia coccinea* Buc’hoz ex Etl. (scarlet sage). It is an herbaceous annual or perennial plant, probably native to Mexico, with paired aromatic leaves and red flowers in whorls, forming simple or branched racemes. This species is an excellent ornamental plant for public green areas and private gardens, and is moderately tolerant to salinity [28,29]. *S*. *coccinea* is described in the scientific literature as a rich source of antioxidant polyphenols with therapeutic potential [30,31,32]. However, to our knowledge, the effects of growing conditions on the antioxidant activity in *S. coccinea* remain unknown. Likewise, no information on growth parameters or physiological response of *S. coccinea* to exogenous SA has been published. Thus, the present study aimed to investigate the effects of applying SA and sodium chloride on growth, pigments content, total polyphenols, and antioxidant activity of *S. coccinea*.

## 2. Results and Discussion

### 2.1. Growth Attributes 

**Figure 1:**
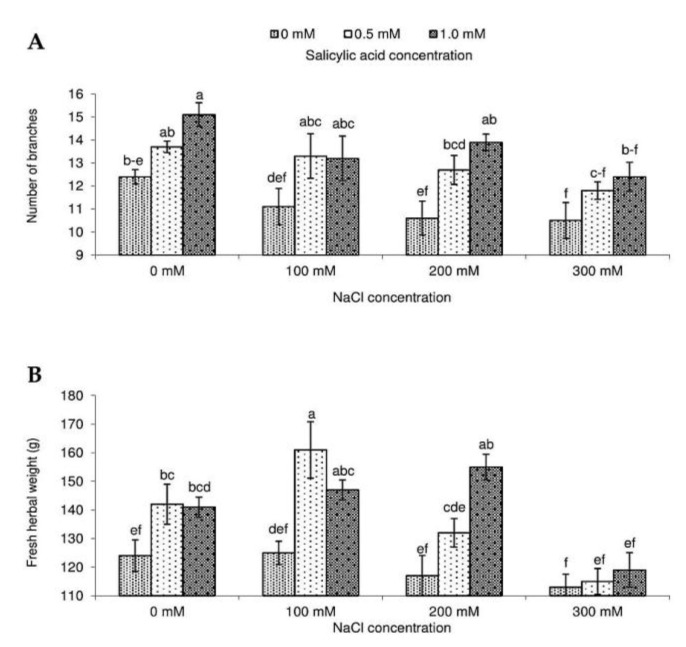
Effect of different levels of sodium chloride (NaCl), salicylic acid (SA), and interaction between NaCl and SA on the number of branches (**A**) and fresh herbal weight (**B**) of *Salvia coccinea*. Bars within a chart with the same lower-case letter are not significantly different at *p* ≤ 0.05. Values represent the means of three replications ± standard deviations.

Salt stress affects all major processes and plant metabolic pathways and reduces plant quality. In this study, salt stress at 300 mM NaCl negatively affected the number of branches and fresh weight of the aboveground parts of a plant determined at the beginning of flowering (fresh herbal weight) (Figure 1). 

Moderate salinity (25–75 mM) was reported to stimulate plant growth in *S. coccinea*, but high NaCl concentration negatively affected morphological features [29]. Exogenous application of SA increased the number of branches and fresh herbal weight under non-stressed conditions as compared to control plants. Increased biomass resulting from SA treatment was also reported in other species such as pepper [21], ginger [33], and artichoke [25]. The growth-promoting effects may be due to SA-mediated enhancement of the efficiency of photosynthesis, transpiration, and uptake of nutrients [34]. SA may also influence plant hormonal status and improve plant growth by controlling their metabolism [26,35]. Our study showed the highest fresh herbal weight in plants treated with 0.5 mM SA and 100 mM NaCl. This confirms previous reports that adverse effect of salt stress on plant growth may be effectively reduced by exogenous SA [23,36,37]. 

### 2.2. Photosynthetic Pigment Contents 

**Figure 2:**
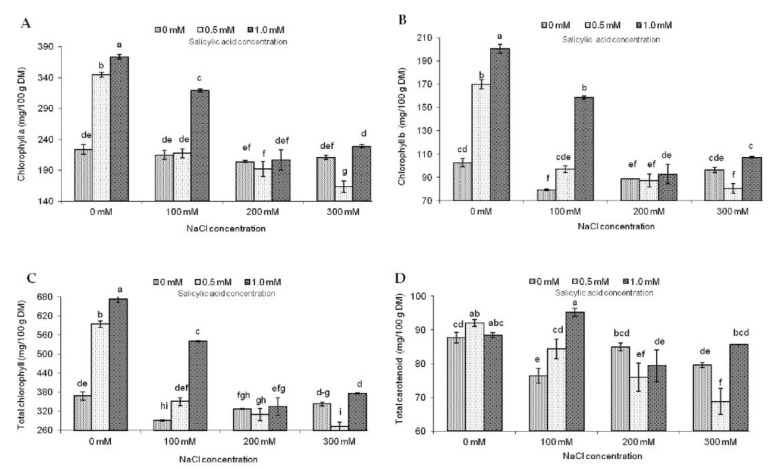
Effect of different levels of sodium chloride (NaCl), salicylic acid (SA), and interaction NaCl and SA on chlorophyll *a (***A**), chlorophyll *b* (**B**), total chlorophyll (**C**), and total carotenoids (**D**) of *Salvia coccinea*. Bars within a chart with the same lower-case letter are not significantly different at *p* ≤ 0.05. Values represent the means of three replications ± standard deviations.

Salt stress inhibits chlorophyll and total carotenoid concentrations in the leaves of many crop plants [38]. According to Gengmao et al. [39], the content of photosynthetic pigments in *Salvia miltiorrhiza* leaves decreased with increasing salinity. It is also visible in the results of our study. The content of chlorophyll *a*, *b*, total chlorophylls, and total carotenoids decreased significantly with increasing salinity (Figure 2). 

SA treatment induces an increase in photosynthetic efficiency in chloroplast, resulting in higher content of chlorophylls in plant tissues [22]. Rady and Mohamed [17] reported that SA significantly enhanced the content of these pigments in the leaves of common bean plants grown in moderately salinized soil. In our study, SA significantly increased chlorophyll *a* and total chlorophyll content in plants not exposed to NaCl (Figure 2). For salinized soil, the most effective SA concentration was 1.0 mM, and it was especially evident when the concentration of NaCl was 100 mM. Similar relations occurred for chlorophyll *b*. However, there were no significant differences in chlorophyll *b* content between plants treated with 0 mM NaCl + 0.5 mM SA, and 100 mM NaCl + 1.0 mM SA. 

**Figure 3:**
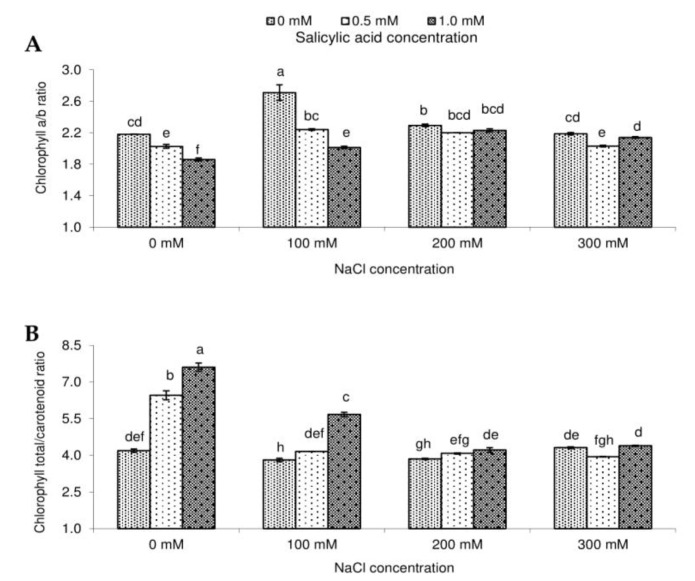
Effect of different levels of sodium chloride (NaCl), salicylic acid (SA), and interaction between NaCl and SA on the ratio of chlorophyll *a* to chlorophyll *b* (**A**), and the ratio of total chlorophyll to carotenoids (**B**) of *Salvia coccinea*. Bars within a chart with the same lower- case letter are not significantly different at *p* ≤ 0.05. Values represent the means of three replications ± standard deviations.

Chlorophyll *a*/*b* ratio was the lowest for the control object, and it dropped with growing salinity (Figure 3). As for the effect of SA concentration on chlorophyll *a*/*b* ratio, the highest value was noted for the control, and it diminished with raising SA concentration. Moreover, an interaction between the analyzed experimental factors showed the highest chlorophyll *a*/*b* ratio for 100 mM NaCl + 0 mM SA and the lowest for 0 mM NaCl + 1.0 mM SA. Similarly as for chlorophylls, the highest carotenoid content transpired in the variant without NaCl treated with 0.5 mM SA (Figure 2). The most evident effect of SA on the content of total carotenoids in plants grown in salinized soil was detected for the combination 100 mM NaCl + 1.0 mM SA. An analogous situation was noted for the chlorophyll/carotenoid ratio (Figure 3). Some authors reported that lowering of chlorophyll *a* to chlorophyll *b* ratio indicates photoinhibition due to damage of photosystem II [40]. Moreover, the changes in the chlorophyll total to carotenoids ratio may be one of the symptoms of oxidative stress [41]. 

### 2.3. Total Polyphenol Content and Antioxidant Activity 

**Figure 4:**
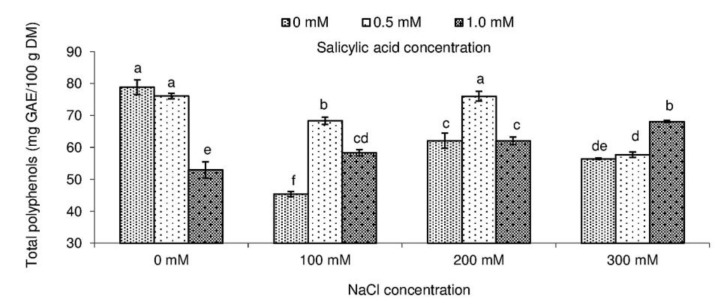
Effect of different levels of sodium chloride (NaCl), salicylic acid (SA), and interaction between NaCl and SA on total phenolic content of *Salvia coccinea*. Bars within a chart with the same lower-case letter are not significantly different at *p* ≤ 0.05. Values represent the means of three replications ± standard deviations.

**Figure 5:**
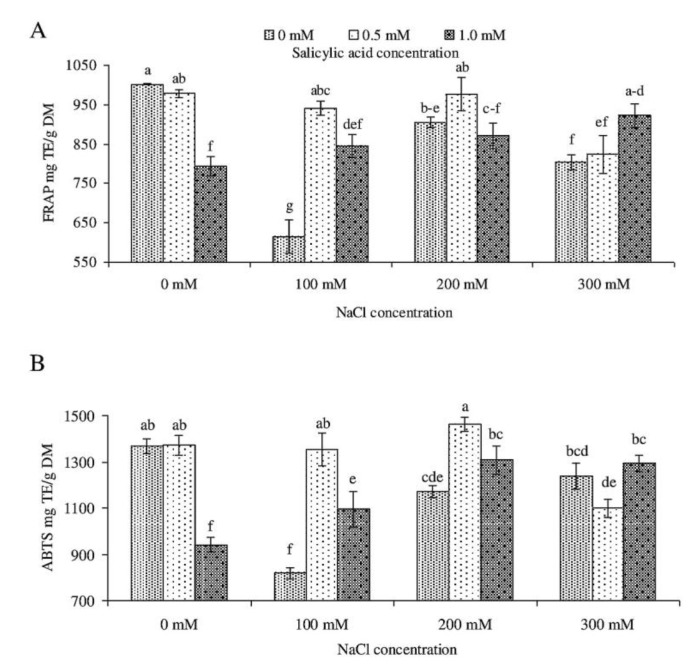
Effect of different levels of sodium chloride (NaCl), salicylic acid (SA), and interaction between NaCl and SA on antioxidant activity of *Salvia coccinea* through ferric-reducing ability of plasma (FRAP) scavenging activity (**A**), and 2,2′-azino-bis(3-ethylbenzothiazoline-6-sulfonic acid) diammonium salt (ABTS) (**B**) assays. Bars within a chart with the same lower-case letter are not significantly different at *p* ≤ 0.05. Values represent the means of three replications ± standard deviations.

Although SA and salt stress significantly affected leaf polyphenol content and antioxidant activity of *Salvia coccinea* (Figure 4 and Figure 5). The content of total polyphenols was the highest (78.81 mg GAE/g DM) in the leaves in the control object and the lowest (45.38 GAE/g DM) in the plants watered with 100 mM NaCl and not treated with SA. FRAP assay used for determining antioxidant activity in *S. coccinea* leaves treated with NaCl and SA showed the highest values (1001.94 mg TE/g DM) in the control plants. We detected the greatest antioxidant potential (1461.56 mg TE/g DM) also assessed by FRAP in the plants treated with 200 mM NaCl and 0.5 mM SA.

The results which we obtained in total polyphenols were higher compared with the results of Muráriková et al. [9], who reported polyphenol content of 34.08 mg GAE/g DM in *S. coccinea* plants. In the study of Bilgin et al. [32], total polyphenol content in *S. coccinea* leaves ranged from 3.48 (50% MeOH) to 43.18 mg GAE/g DM (100% MeOH), depending on the hydroalcoholic solvent ratio. Pop et al. [1] used two different methods of plant extraction and reported the values to change from 24.90 to 36.72 mg GAE/g DM in the leaves of *Salvia officinalis* (‘Tricolor’ and ‘Purpurascens’), from 19.84 to 28.90 in *Salvia lavanduifolia*, and 7.68 to 11.23 in *Salvia elegans*. Tehami et al. [42] found that total polyphenols in the leaves of *Salvia argentea* amounted to 71.80 mg GAE/g DM (aqueous extract) and 87.13 mg GAE/g DM (methanolic extract). Lamien-Meda et al. [3] expressed total polyphenol content in *S. officinalis* aerial parts in mg of catechin equivalents that varied from 50 to 89 mg CAE/g DM. According to Lamien-Meda et al. [3] and Farhat et al. [4], the quantitative variations of polyphenols in the plant material may be due not only to the extraction and quantification methods, but also to geographical or climatic factors, vegetative phase, genotypes, etc. Moreover, polyphenol synthesis and their accumulation are generally stimulated in response to biotic/abiotic stresses, such as salinity [43,44]. Valifard et al. [45] showed that both phenolic content and antioxidant activity in *S. mirzayanii* leaves increased until mild salinity stress (6.8 dS/m), but declined at high salinity (9.1 dS/m). Also, Taârit et al. [44] reported that at 25 and 50 mM NaCl total polyphenol content and antioxidant activity were higher than in control plants but declined with an increase in NaCl from 50 to 75 mM. Our results show the same tendency for total polyphenol content, FRAP, and ABTS antioxidant activity with increasing salinity. However, at the highest NaCl concentration (300 mM), these parameters were lower than at 200 mM NaCl (Figure 4 and Figure 5).

Plants exposed to 100 mM NaCl and sprayed with SA at both concentrations (0.5 and 1.0 mM) showed significantly higher total phenolic content, as well as leaf antioxidant activity, as determined by both FRAP and ABTS. Also, Thiruvengadam et al. [46] reported that plant treatment with salicylic acid results in higher total polyphenol content. Results from this experiment clearly indicated that exogenous SA increases antioxidant activity in plants under moderate salinity levels. The outcomes of our study corroborate recent findings that high antioxidant activity in stressed plants is a result of increasing activity of antioxidant enzymes that mitigate the negative effect of reactive oxygen species [13,47].

## 3. Materials and Methods

### 3.1. Plant Culture, Treatment, and Measurement of Growth Parameters 

Seeds of *Salvia coccinea* were obtained from Breeding and Seed Company W. Legutko (Jutrosin, Poland). Before germination, the seeds were surface-sterilized in 0.2% sodium hypochlorite solution for 10 min, and then rinsed several times with distilled water. They were sown on 30th March 2014 into boxes filled with substrate base (Klasmann-Deilmann, Geeste, Germany), in controlled-environment glasshouse (21/17 °C day/night). After three weeks, uniform seedlings were transferred into pots 8 cm in diameter, filled with TS1 plant substrate (Klasmann-Deilmann). On 28 May 2014, single seedlings were transferred into black plastic pots of 17 cm in diameter and 2 dm^3^ capacity. The substrate was deacidified peat at pH 6.5, mixed with Yara Mila Complex fertilizer (Yara International ASA, Oslo, Norway) containing 12% N, 11% P_2_O_5_, 18% K_2_O, 2.7% MgO, 8% S, 0.015% B, 0.2% Fe, 0.02% Mn, and 0.02% Zn, used at 2.5 g/dm^3^. The pots were transferred to a non-heated tunnel covered with double layer of plastic located in the area of West Pomeranian University of Technology in Szczecin (53°25′ N, 14°32′ E; 25 m a.s.l.). Mean monthly air temperature inside the tunnel was May 18.9 °C, June 19.7 °C, July 24.1 °C, and August 19.6 °C.

Salicylic acid SA (Sigma Aldrich, St. Louis, MO, USA) was dissolved in a small amount of ethanol (Chempur, Piekary Śląskie, Poland). The plants were sprayed early in the morning with 0.5 or 1 mM SA three times, every seven days, starting from 1 June 2014. Each time individual plants were sprayed with about 20 cm^3^ of the solution. The solution was supplemented with Silwet Gold wetting agent (Momentive Performance, Wilton, CT, USA). The plants exposed to salt stress were irrigated with a solution of sodium chloride NaCl (Chempur, Poland), four times, every five days from 1 July 2014, using 250 mL of the solution per plant per application. The following NaCl concentrations were used: 100, 200, and 300 mM. Control plants were irrigated with tap water with electrical conductivity (EC) 0.25 mS·cm^−1^. Each treatment had four replicates, and each replicate included five plants. On 20 September 2014, at the beginning of flowering, the plants were harvested for determination of growth parameters. The number of branches and fresh herbal weight, e.g., the total fresh weight of the aboveground parts of a plant (leaves, stems, and inflorescences) were recorded. Leaves were dried for four weeks at 25–30 °C, then placed into paper bags and stored at room temperature in the dark until use. Chemical analyses of dried material were performed within two months from harvest.

### 3.2. Photosynthetic Pigment Determination

The content of chlorophylls and carotenoids was determined using a spectrophotometric method described by Lichtenthaler and Wellburn [48] with some modifications. The weighted (with an accuracy of 0.0001 g) samples of ground dried leaves (about 0.5 g) were triturated in the presence of 80% (*v*/*v*) aqueous acetone, and transferred in 80% acetone to 50 cm^3^ volume measuring flask. The flasks with the samples were placed in an ultrasonic cleaner for 5 min. Then the extract samples (7 mL) were centrifuged at 13,000 rpm for 10 min. The supernatant was transferred to a cuvette, and the absorbance was measured at 441, 646, 652, and 663 nm using a Helios Gamma Spectrophotometer (Thermo Spectronic, Cambridge, UK).

Equations used for the calculations are presented below:

Chlorophyll a = 12.21 × E_663_ − 2.81 × E_646,_ (1)

Chlorophyll b = 20.13 × E_646_ − 5.03 × E_663,_ (2)

Total chlorophyll = 27.8 × E_652,_ (3)

Total carotenoids = (1000 × E_441_) − 3.27 × (12.21 × E_663_ − 2.81 × E_646_) − 104 × (20.13 × E_646_ − 5.03 × E_663_). (4)

Chlorophyll and carotenoid contents were expressed in mg/100 g of dry matter after correction for moisture content (drying the samples at 105 °C to constant weight). Each analysis was carried out in triplicate.

### 3.3. Determination of Total Polyphenol Content and Antioxidant Activity

#### 3.3.1. Preparation of Plant Extracts

Plant extracts used for determination of total polyphenol content and antioxidant activity were reported as proposed by Wojdyło et al. [49], with some modifications as described in the previous work [50]. A sample of 1 g dried ground leaves of *Salvia coccinea* was treated with 80% methanol (MeOH) to a final volume of 100 mL. The mixtures were ultrasonicated for 30 min (2 × 15 min) and then left to stand for 24 h at room temperature (~20 °C). The resulting extracts were filtered through Whatman No. 1 filter paper. Then, the filtrates were centrifuged for 10 min at 1500 rpm. All the extractions were prepared in triplicate. The extracts were kept at 4 °C and analyzed within 24 h.

#### 3.3.2. Total Polyphenol Content

Content of total polyphenols was analyzed spectrophotometrically using the Folin–Ciocalteu colorimetric procedure, according to the method described by Wojdyło et al. [49]. The plant extract (100 µL), 0.2 mL of Folin–Ciocalteu reagent, 2 mL of deionized water, and 1 mL of 20% sodium carbonate, were mixed and incubated for 1 h at room temperature in darkness. Then, absorbance was measured at 760 nm and gallic acid (GAE) was used for calibration of the standard curve. Total polyphenols were calculated as mg GAE equivalent per g of dry matter sample (DM).

#### 3.3.3. Determination of Ferric-Reducing Antioxidant Power (FRAP)

The antioxidant activity of the samples was also determined using the ferric-reducing ability of plasma FRAP method reported by Wojdyło et al. [49]. FRAP reagent was prepared freshly by mixing acetate buffer (300 mM, pH 3.6), a solution of 10 mM TPTZ (2,4,6-tris(2-pyridyl)-s-triazine) in 40 mM HCl, and 20 mM FeCl_3_·6H_2_O (iron(III) chloride hexahydrate) at 10:1:1 *(v*/*v*/*v*), and warming it at 37 °C before use. For the determination of antioxidant activity, 2.7 mL of FRAP reagent was mixed with 0.3 mL of the sample solution. The absorbance was measured spectrophotometrically at 593 nm after four min. The standard curve was prepared using different Trolox concentrations. FRAP activity was calculated as mg TE per g DM.

#### 3.3.4. Determination of Free Radical-Scavenging Ability Using a Stable ABTS Radical Cation

Free radical-scavenging activity was carried out according to the ABTS radical cation decolorization procedure reported by Chew et al. [51], Wojdyło et al. [49], and Re et al. [52] with some modifications as described in the previous work [50]. ABTS (2,2′-azino-bis(3-ethylbenzothiazoline-6-sulfonic acid) diammonium salt was dissolved in deionized water to a 7 mM concentration. ABTS radical cation (ABTS^•+^) was generated by mixing ABTS stock solution with 2.45 mM potassium peroxodisulfate. The reagent was kept in the dark at room temperature for 16 h before using. Subsequently, the ABTS^•+^ solution was diluted with PBS (phosphate buffered saline, pH 7.4) to an absorbance of 0.7 (±0.02) at 734 nm before usage. The reaction was initiated by adding 3 mL of diluted ABTS^•+^ solution (A_734_ = 0.7 ± 0.02) to 300 µL of methanolic plant extracts. The absorbance was measured at 734 nm, exactly 6 min after mixing. The results were calculated from the calibration curve using Trolox as standard, and expressed as mg TE per g dry weight sample. 

### 3.4. Statistical Analysis

The experiment was set up in the system of complete randomization. The results were statistically analyzed by means of analysis of variance for 2-factor experiments using STATISTICA 13.0 software (Statsoft, Cracov, Poland). After checking the good fit of the model, post hoc comparison was done using the Tukey’s multiple range test.

## 4. Conclusions

*Salvia coccinea* is a promising source of potential antioxidants, and its extracts can be used in the pharmaceutical industry, as well as in food products and cosmetics. *S. coccinea* plants responded positively to salicylic acid (SA) application. SA stimulated the growth of plants via improved the number of branches, fresh herbal weight, and total chlorophyll content. Salt stress reduced growth, leaf pigment content and total polyphenols in plants. However, foliar application of SA relieved the adverse effects of 100 mM NaCl, as demonstrated by increased biomass, content of photosynthetic pigments, and total polyphenols, as well as antioxidant potential by FRAP and ABTS. 

So, we do agree with the statement that salt-stressed plants might represent potential sources of phenolic compounds for economical use. Additional SA treatment may further increase their content in plants grown under mild salinity stress and also enhance plant antioxidant activity and growth parameters.
